# Reply to Takeshita, W.M.; Ribeiro, D.A. Comment on “Pop et al. Early Diagnosis of Oral Mucosal Alterations in Smokers and E-Cigarette Users Based on Micronuclei Count: A Cross-Sectional Study among Dental Students. *Int. J. Environ. Res. Public Health* 2021, *18*, 13246”

**DOI:** 10.3390/ijerph19073845

**Published:** 2022-03-24

**Authors:** Anca Maria Pop, Raluca Coroș, Alexandra Mihaela Stoica, Monica Monea

**Affiliations:** 1 Faculty of Medicine, George Emil Palade University of Medicine, Pharmacy, Science, and Technology of Târgu Mureș, 540139 Tirgu Mures, Romania; ancapop98@yahoo.com; 2 Faculty of Dental Medicine, George Emil Palade University of Medicine, Pharmacy, Science, and Technology of Târgu Mureș, 540139 Tirgu Mures, Romania; raluca_coros@yahoo.com; 3 Department of Odontology and Oral Pathology, George Emil Palade University of Medicine, Pharmacy, Science, and Technology of Târgu Mureș, 540139 Tirgu Mures, Romania; alexandra.stoica@umfst.ro

Thank you for your interest [1] in our paper [2]. Below we tried to provide answers to your observations regarding our research.

The use of Papanicolaou (Pap) stain is a valuable option for screening, as it is more accessible than DNA-specific stains. Moreover, we took into consideration criteria for differential diagnosis with several confounding factors, such as bacteria (which can be present not only in the proximity of the nucleus) or cytoplasmatic granules (numerous bodies with different localizations, which do not comply with the criteria proposed for micronucleus identification) [3].

Regarding the number of micronuclei (MN) found in the control group, we want to highlight the following aspects: as stated by Bonassi et al. [4] in the reference you cited, the authors considered as a normal range a number of micronucleated cells (MNC)/1000 buccal exfoliated cells between 0.32–1.7‰. In our study, the value of MNC/1000 buccal exfoliated cells was 1.4‰, which falls in the aforementioned range. However, according to Figure 2 from the same article, the values of MNC in healthy subjects showed a wider distribution. Our aim was not to find normal limits from such a small sample but to present the results obtained from a group of participants who never smoked. Other factors such as lifestyle or environmental exposure were not taken into consideration. Furthermore, the sampling protocol included the use of a spatula, which is recognized even in the reference you provided [4] as giving higher numbers of MN. The spatula also shows a higher sensitivity in detecting alterations of the oral mucosal cells compared to other sampling tools, such as a cytobrush [5]. From a clinical point of view, we would be more concerned about false-negative results, because these would be more detrimental to patients’ health compared to false-positive ones. We acknowledge that the specimens with higher values of MN should be further evaluated by more specific techniques.

Regarding the figures and tables, they are, in our opinion, clear, self-explanatory and easy to follow if one also reads the information presented in the Material and Methods section (the description of Groups A, B and C and the abbreviations MN and MNC). The same information should not be repeated in different parts of the article, as also suggested by the guidelines of the journal. In order to answer your observation upon the reporting of both MN and MNC we present a paragraph from the study used as the reference for this approach: “The frequency of micronucleated cells should be used as the main endpoint; however, the scoring of the number of micronuclei may also be used as a separate measure to show whether cells with multiple micronuclei were present” [6]. This approach is also encouraged by other researchers [7].

We decided to evaluate 1000 cells based on a review published in 2021 [8], which included eight studies of oral or head and neck cancers conducted between 2012–2019, after the publishing of the article you suggested as reference and seven of them used ≤1000 cells for MN scoring. Your observation regarding the misuse of the term “cytotoxicity” instead of “genotoxicity” is correct, but we consider that this does not alter the message we wanted to highlight. We agree that the scoring of all the parameters included in the buccal micronucleus cytome assay is important; however, we chose to report the number of MN as, based on the same recent review [8], this was the only parameter showing a positive correlation with the severity of the disease: “The results on biomarkers other than the MN showed some inconsistency among the studies. A common result was that the nuclear alterations associated with apoptosis (karyorrhexis, condensed chromatin, and pyknosis) occurred less frequently in cancer patients with respect to the controls and in cells from lesion area than in cell from unaffected mucosa” [8].

We hope that this topic will remain interesting for many researchers in order to provide more information regarding the effects of e-cigarettes and better preventive measures for oral cancer. We found your observations very valuable for developing future research protocols.

## Data Availability

Not applicable.
